# Local Sustainability and Gender Ratio: Evaluating the Impacts of Mining and Tourism on Sustainable Development in Yunnan, China

**DOI:** 10.3390/ijerph120100927

**Published:** 2015-01-19

**Authors:** Ganlin Huang, Saleem Ali

**Affiliations:** 1 State Key Laboratory of Earth Surface Processes and Resource Ecology (ESPRE), Center for Human-Environment System Sustainability (CHESS), Beijing Normal University, Beijing 100875, China; 2 Centre for Social Responsibility in Mining (CSRM), Sustainable Minerals Institute, The University of Queensland Brisbane, Queensland 4072, Australia; E-Mail: s.ali3@uq.edu.au; 3 Gund Institute for Ecological Economics, Rubenstein School of Environment and Natural Resources, University of Vermont, Burlington, VT 05401, USA

**Keywords:** sustainable development, gender ratio, mining, tourism, China

## Abstract

This study employed rapid evaluation methods to investigate how the leading industries of mining and tourism impact sustainability as manifest through social, economic and environmental dimensions in Yunnan, China. Within the social context, we also consider the differentiated impact on gender ratio—which is a salient feature of sustained development trajectories. Our results indicate that mining areas performed better than tourism areas in economic aspects but fell behind in social development, especially regarding the issue of gender balance. Conclusions on environmental status cannot be drawn due to a lack of data.  The results from the environmental indicators are mixed. Our study demonstrates that rapid evaluation using currently available data can provide a means of greater understanding regarding local sustainability and highlights areas that need attention from policy makers, agencies and academia.

## 1. Introduction

Sustainable development (SD) has been defined in terms of intergenerational equity for the past 30 or so years as “development which meets the needs of the present without compromising the ability of future generations to meet their own needs” [1]. Its definition, policy application and evaluation frameworks have been debated by researchers and practitioners (e.g., [2,3,4,5,6,7]). While numerous studies have assessed sustainability for various development programs, relatively few have looked at the relationship between two leading industries and sustainability. This study aims to investigate the impact of two leading industries on SD through a case study in China. Our aim in this study is also to compare a primary industry (mining) with a service industry (tourism). Such a comparison has two key empirical objectives. The first objective is to explore the feasibility of a rapid spatial evaluation and assess what information it can provide to decision-makers. The second objective is to draw preliminary conclusions from the case study in China on how the leading industries of mining and tourism may impact sustainability. 

There have been extensive debates surrounding the definition and implementation of SD. Since 1990, researchers have been trying to reach a consensus on an explicit and implementable definition for SD (e.g., [8,9,10]). After discussions lasting over three decades, it is generally agreed that the concept of SD is highly dynamic [11,12,13], largely indefinite [14,15,16] and highly contested [9,17]. Although no consensus has been reached on a specific definition of SD, it is generally agreed that SD encompasses the economic, environmental and social dimensions of the development process [2,18].

Sustainable development has been widely accepted and used by development planners, environmental activists and researchers. It has been increasingly accepted as a fundamental objective for development programs designed by environmental organizations (such as the World Wildlife Fund) and financial institutions (such as the World Bank), and for public policy in many countries [7]. Therefore, evaluating program and policy achievements from the perspective of SD becomes an important issue. Various indicator systems have been designed to assess SD programs and projects (e.g., [5,6]). Beyond project level assessments, it has been suggested that there is a need to integrate the three dimensions of SD (economic, social and environmental) into a comprehensive assessment [2].

While these evaluation frameworks and indicator systems are important tools to understand how SD is implemented, using these tools requires many resources including data and technical skills. Consequently, many evaluation frameworks or indicator systems are only available to researchers and agencies with adequate funding and personnel resources. Local governments need to understand local sustainability and the impact from leading industries; however, they may not be able to benefit from these tools due to limited resources. 

This study aims to fill this gap through a case study in Yunnan, China. Based on secondary data, we evaluated and compared SD progress between areas that are dominated by mining and those dominated by tourism in Yunnan. Specifically, we ask the question whether mining areas and tourism areas in Yunnan perform differently in economic, environmental and social dimensions. 

The development impact of mining has been widely contested because it is an inherently obsolescent sector in which a nonrenewable resource is extracted. However, mining can lead to numerous downstream opportunities if resource rents can be properly managed [19]. As a sector often dominated by men, mining has historically contended with gender equity challenges, even in countries where legal protection is offered against gender discrimination [20]. Tourism offers more employment opportunities for both genders but is also highly vulnerable to shocks due to natural disasters or political instability. Although these features of both sectors are well-known, the demographic dimensions of such features have not been empirically examined in areas of coexistence of mining and tourism sectors in the way we have done using a rapid evaluation technique.

## 2. Study Site

Yunnan province is located in the southwest corner of China bordering the countries of Vietnam, Laos, and Burma. Yunnan is approximately 393,898 square kilometers and has a population of 44.5 million people [21]. 

Yunnan has vast mineral and tourism resources. Both industries were supported by local governments to stimulate local development and have experienced dramatic growth over the past twenty years. In 2006, Yunnan had more than 7000 mining enterprises. The mining industry extracts and produces more than 80 mineral resources, including coal, iron, manganese, copper, lead, zinc, tin, gold, phosphorus and cement. The industry plays a prominent role in the local economy, employing about 350,000 workers, which is 10% of total employment [21]. The total production value of mineral exploration was 7.5 billion USD in 2006. The mining industry contributed to a third of the industrial added value in 2006, which was 21.4 billion USD, equivalent to 42.7% of Yunnan’s gross domestic production (GDP) [21]. 

**Figure 1:**
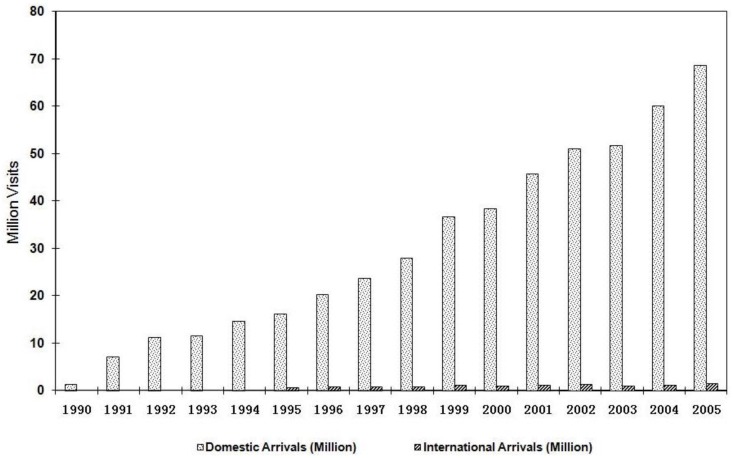
Tourism growth in Yunnan.

The tourist industry has been growing steadily in Yunnan since 1990. Domestic tourism income has been increasing at an average annual rate of 347.9% during 1990–2005. In 2006, tourism generated 6.3 billion USD. Tourism income contributed 32.4% to the service sector added value, which was 19.3 billion USD, equivalent to 38.5% of Yunnan’s GDP [21]. Figure 1 shows domestic and international tourism development in Yunnan from 1990 to 2005 [21,22].

While both mining and tourism play an important role in the local economy, their contributions to SD remain unclear. Our research has previously identified overlapping spatial patterns of mining and tourism, as they relate to biodiversity in Yunnan [23]. We note that urban planners, particularly in developing countries, have limited data to inform land use decisions, and the kind of rapid evaluation approach provided here has potential for improving the speed and efficacy of decision-making. This study conducted a rapid SD evaluation between areas dominated by mining and those dominated by tourism to explore mining and tourism’s impacts on socioeconomic and environmental variables.

## 3. Method

### 3.1. Data

**Figure 2:**
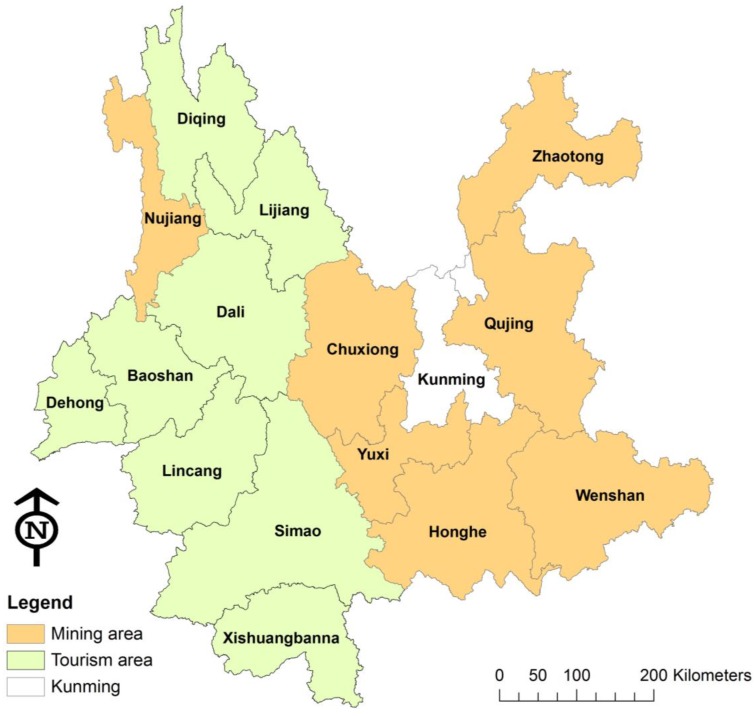
Mining and tourism areas in Yunnan.

Prefecture, the administrative unit under province, was used as the unit of analysis in this study. There are 16 prefectures in Yunnan province (Figure 2), which were recognized as mining areas or tourism areas based on mining production value, tourism income [21] and interviews with local officials in mining and tourism departments. Figure 1 shows how the 16 prefectures were grouped as mining areas, tourism areas, and areas where neither industry dominated. 

#### 3.1.1. Economic Status

Economic status of each prefecture was defined by GDP and the percent of counties living below the poverty line. Gross domestic production is the primary economic indicator and describes overall economic development. It was obtained from the Yunnan Statistic Bureau [21]. 

Whether a county is in poverty is evaluated and determined by the central government. The primary indicator of poverty is the average annual household income with a threshold varying from 50 to 87.5 USD. Other factors, including wealth distribution, geographic location, and economic structure are also considered. Therefore, high percentage of counties in poverty not only indicates lower incomes, but also reflects the poor conditions of transportation, low literacy, little social welfare, and a lack of income sources to improve economic situations. The percent of counties in poverty within a prefecture is from the Yunnan poverty reduction office [24].

#### 3.1.2. Social Development

Studies have shown that economic indicators (such as GDP or poverty rate) alone do not adequately measure social well-being or quality of life [25]. Therefore, this study incorporated three other indicators to describe the overall socioeconomic development, including: (1) access to major roads, (2) access to clean water, and (3) gender ratio. Access to transportation networks and clean water is crucial to people’s quality of life. The distribution of these services in a prefecture reflects its overall development. Percent villages having access to major road and clean water were obtained from the Yunnan Statistical Bureau [21]. 

Gender ratio is another important indicator for overall social development [26,27]. Women tend to live longer than men when receiving similar nutritional attention and health care [27]. The gender ratio (defined as female/male ratio) is around 1.05 or 1.06 in Europe, North America and Japan [27]. In contrast, the ratio is 0.94 or even lower in South Asia, West Asia and China [27]. The deficit of women indicates inequality and neglect leading to excess mortality of women. Altered gender ratios may cause various social problems such as “marriage squeeze” [26] Economic growth alone may not improve living conditions for women or have any significant effect on gender imbalances [27]. The deficit of women has to be addressed by economic, social and cultural factors and therefore is a good indicator of overall social development in an area. Data on gender ratios were also obtained from the Yunnan Statistical Bureau [21].

#### 3.1.3. Environmental Status

Three indicators: ecological index, river water quality, and forest coverage, were used to evaluate environmental status. Ecological index for each prefecture was calculated based on the ecologically integrated appraisal indicators system [28]. This system uses a number of indicators, including forestry, farm land, water quality, land erosion, natural disasters, noise, waste, air quality and population density, to assess the overall ecological and environmental health for each county in Yunnan [28]. The ecological index ranges from 0 to 1, with 0 indicating the worst conditions. Given that prefecture was our unit of analysis, ecological index was calculated for each prefecture as an average of the ecological indices of all the counties within that prefecture. 

Water quality of a prefecture was calculated as an average of river water quality weighted by the river length for all the rivers within the prefecture boundary. Data of river water quality and river length were obtained from the Center of Yunnan Environment Information [29]. There are five categories of river water quality, with 1 being the least polluted and 5 as the most polluted according to the regulation of environmental quality assessment [30].

Forest coverage in prefectures was obtained from an online database called “*Chinese natural resources, environment, economic and population database*” [31]. The forest coverage data came from a national survey conducted in 1998. The data measures the percent area of land that is covered by forest.

#### 3.1.4. Temporal Dimension of Data and Time-Series Analysis

The data in this study were collected at different years including 1998, 1999, and 2005 (Table 1). This is primarily due to limited data availability. Environmental monitoring, such as the forest coverage survey, is carried out once every several years. In order to make the best use of available data sets, we used all the data sets and conducted a two-phase time-series analysis. Forest coverage, which was surveyed in 1998, was considered along with other data collected in 1999 as the first time phase. Data collected in 2005 were considered as the second time phase.

**Table 1:** Indicators of economic development, environmental status and social development.

Item	Prefecture	Source	Year 1	Year 2
Economic Development	GDP per capita	[21]	1999	2005
	Pct Poor Counties	[24]		2005
Environmental Status	Prefecture ecological index	[28]	1999	
	Water quality index	[29]		2005
	Forest coverage	[29]	1998	
Social Development	Pct villages having access to major roads	[21]	1999	2005
	Pct villages having access to pipe water	[21]	1999	2005
	Gender ratio	[21]	1999	2005

### 3.2. Data Analyses

First, we calculated and compared the means of all the indicators in mining and tourism areas. Then, two-sample *t*-tests were conducted in SPSS to examine whether the indicators were statistically different in mining areas compared to tourism areas. A *t*-test assesses whether the means of two groups are statistically different from each other. Typically, the values of each indicator will differ from site to site, and therefore the two groups are unlikely to have the same distributions or means. A *t*-test is used to determine whether the difference between the two groups is larger than variances within groups. If the variance between groups is larger than the variance within groups, the two groups are considered statistically different.

## 4. Results 

Mining areas had a higher mean value of GDP and a lower mean value of poverty rate than tourism areas. During 1999–2005, GDP grew dramatically in both mining (58.95%) and tourism areas (80.15%, see Table 2). Poverty rates were only available for 2005 and therefore cannot predict trends.

**Table 2:** Comparing mining areas and tourism areas.

Item	Leading Industry	1999	2005	Change
GDP per capita	Mining areas	4597.39	7307.42	58.95%
	Tourism areas	3119.239	5619.44	80.15%
Pct Poor Counties	Mining areas	--	37.33	--
	Tourism areas	--	47.88	--
Pct villages having access to major roads	Mining areas	89.20	97.60	8.40%
	Tourism areas	92.51	98.23	5.72%
Pct villages having access to pipe water	Mining areas	69.15	87.10	17.95%
	Tourism areas	76.98	91.53	14.55%
Gender ratio	Mining areas	0.9355	0.9346	0.09%
	Tourism areas	0.9515	0.9533	−0.19%
Prefecture ecological index	Mining areas	446.78	--	--
	Tourism areas	535.43	--	--
River water quality index	Mining areas	--	3.48	--
	Tourism areas	--	3.25	--
Forest coverage	Mining areas	17.78	--	--
	Tourism areas	13.53	--	--

According to social indicators, mining areas experienced less social development compared to tourism areas. Mining areas had lower values in all three social indicators (*i.e.*, gender ratio, percent of villagers with access to clean water and roads) suggesting mining areas had less accessibility to major roads and clean water, and fewer females proportionally compared to tourism areas. From 1999 to 2005, the accessibility of major roads and clean water increased in both mining and tourism areas (Table 2). Percent village having access to major roads grew 8.40% in mining areas and 5.72% in tourism areas. Percent village having access to clean water grew 17.95% in mining areas and 14.55% in tourism areas. It is worth noting that mining and tourism areas experienced different trends in gender ratios. While the gender ratio *decreased* from 0.9355 to 0.9346 from 1999 to 2005 in mining areas, it *increased* from 0.9515 to 0.9533 in tourism areas. 

Environmental indicators were only available at one time, and thus did not provide enough information to analyze trends. Mining areas had a lower ecological index, indicating worse ecological status compared to tourism areas. Mining areas also had a slightly higher value in river water quality, suggesting worse water quality. Mining areas had higher average forest cover (17.78%) compared to tourism areas (13.53%). 

While the means of the indicators differed between mining and tourism sites, none of them were significant at the 95% confidence level (Table 3). At the 90% confidence level, the 2005 gender ratio (female/male) of tourism areas was significantly higher than mining areas, which means proportionally tourism areas had more women than mining areas and this difference between mining and tourism areas was larger than the variations within mining or tourism areas.

**Table 3:** Results from the two-sample *t*-tests.

Item	Prefecture	1999	2005
Economic Development	GDP per capita	0.386	0.507
	Pct Poor Counties	na	0.196
Environmental Status	Prefecture ecological index	0.208	na
	Water quality index	na	0.418
	Forest coverage	0.270	na
Social Development	Pct villages having access to major roads	0.614	0.270
	Pct villages having access to pipe water	0.299	0.633
	Gender ratio	0.222	0.092 *

Note: ^*^ denotes significant difference evaluated at 90% confidence interval.

## 5. Discussion

### 5.1. Impacts from Mining and Tourism Industries

This study assessed the feasibility of using a rapid evaluation to compare the economic status, social development and environmental situation of mining and tourism areas in Yunnan. Results suggested that on average mining areas are performing better economically compared to tourism areas but fell behind on social development. Conclusions on environmental impacts from the two industries cannot be drawn due to limited data availability. 

Mining areas had higher mean values for GDP in both 1999 and 2005, and a lower mean value for the percent of counties in poverty in 2005. This result is consistent with earlier studies on mining which suggests that mining provides employment opportunities and contributes strongly to the local economy [32]. However, because mining activities extract limited resources relying solely on the mining industry to support a country’s economy may lead to short-term economic gains but limit long-term economic growth, known as “Dutch disease” and “resource curse” (e.g., [33,34]). Although our study did not find that mining areas in Yunnan experienced such economic decline, local governments need to recognize the finite nature of mineral resource and develop other income sources. While having a mean value lower than mining areas, GDP in tourism areas is growing fast. This is consistent with findings from other research on tourism, which demonstrated that tourism tends to grow fast in relatively less developed areas and therefore is an important tool for reducing poverty [35].

When we compared the mean values of the social indicators between mining and tourism areas, we found that the mining areas performed worse compared to tourism areas for all three indicators (Table 2 and Table 3). Although accessibility to clean water and major roads was improving from 1999 to 2005 in both mining and tourism areas, mining areas had less accessibility to both services in 1999 and 2005 compared to tourism areas. The gender imbalance problem was more severe in mining areas than tourism areas in both 1999 and 2005. Many studies have examined various social problems associated with the mining industry including indigenous rights, social justice and sexually-transmitted diseases [13]. In contrast, tourism is usually regarded as a source of income that promotes social development. Our results are consistent with previous findings, suggesting that mining provides less opportunity for social development compared with tourism areas.

Our analysis generated interesting results regarding changes in gender ratio in mining and tourism areas. Gender ratio was the only indicator that diverged between mining and tourism areas when the temporal trend (1999–2005) was examined. While both mining and tourism areas had fewer females than males, mining areas had a lower gender ratio (female/male) than tourism areas in 1999, gained fewer females than males between 1999 and 2005 and resulted in an even lower gender ratio in 2005. In contrast, tourism gained more females than males between 1999 and 2005 and the gender ratio (although still lower than 1) was higher in 2005 than in 1999. This divergent impact on gender ratio should receive attention from policy makers as well as researchers since gender imbalance is already severe in China.

Our results support two earlier findings from studies on gender imbalance. First, economic growth may not promote gender balance [27]. Our study indicates that the richer mining areas (higher GDP and less poverty) experience more severe gender imbalances compared to tourism areas. Furthermore, the deficit of women is deteriorating over time in mining areas while tourism areas are improving on gender imbalance. Secondly, gainful employment for women is necessary to improve gender imbalance [27,36]. Most mining jobs are gender-specific and are not available to women. In contrast, tourism jobs are open to both men and women. Therefore, tourism may contribute to reducing gender imbalance by providing employment opportunities for women. 

It is important to clarify that gender ratio differences between mining and tourism areas are not caused solely by the fact that the mining industry is dominated by male workers. The gender ratio data used in this study is based on registered residency (the “Hu4 kou3” system in China), which does not count migrant workers. In other words, if a person moves to another area to work, his/her residency would not change and he/she would be counted in local population of the hometown instead of where he/she works and lives. 

The results from the environmental indicators are mixed and limited to one point in time. Mining areas had greater forest coverage but worse ecological conditions and river water quality compared to tourism areas. Because all three environmental indicators were only available for one year, it remains unclear whether the differences in environmental indicators between mining and tourism areas existed before the two industries were established.

Results from the two-sample t tests showed that all three indicators were not significantly different between mining and tourism areas at the 95% confidence level. It indicated although the mean values of the indicators are different between mining and tourism areas the variations of the indicators between mining and tourism areas were not larger than those within mining or tourism areas. At the 90% confidence level the difference of gender ratios between mining and tourism areas (*p* = 0.092) became significant. It is worth noting that our sample size for t test was relatively small (*N* = 15), which is more likely to produce a non-significant result.

### 5.2. Advantages and Limitations of the Evaluation Method

The method used in this study has some advantages and can potentially be used as a tool for local governments and NGOs to evaluate local sustainability. Firstly, the analysis uses publicly available datasets, which is important for local governments and NGOs that are interested in understanding local sustainability but do not have the resources to conduct a comprehensive assessment. In addition, these data are typically updated annually (such as indicators from the annual statistic book) or periodically (such as poverty rate, river water quality and forest coverage). Therefore, temporal analyses would not require many additional resources. Secondly, the two-sample t test analysis used here only requires basic statistics skills. As showed in this case study, this analysis provides some important insights on local sustainability with limited datasets. In particular, it provided important insights regarding progress in local areas that are dominated by different industries and highlighted issues that would require attention (*i.e.*, gender ratio in Yunnan’s case). As many areas in China are promoting certain industries to stimulate economic growth, this method may provide an important tool for local government to evaluate how different industries impact local sustainability, thus helping governments make informed decisions. 

This study has some limitations. First, SD has three dimensions: economic, social and environmental. This study did not provide much information on the environmental dimension because the environmental data were not available for temporal analysis. As we discussed above, this study can be easily updated when more data become available. We hope the environmental dimension would be completed after the next survey on river water quality and forest coverage.

Second, our study focused on outcomes and did not address the processes toward SD. Being process-oriented and having meaningful community participation are two important criteria for successful SD [4]. This, however, was not evaluated in our study. Due to limitations of existing datasets our analysis did not provide information on local processes or levels of local involvement in the development. However, our analysis can point out issues that require future research, which may incorporate these criteria. For example, in the case of Yunnan, our study showed that gender ratio differed significantly between mining and tourism areas. This gender imbalance requires further study. Future studies addressing gender imbalances in mining and tourism areas should incorporate both process-oriented approaches and community participation to examine why and how mining and tourism areas had different gender ratios.

## 6. Conclusions

This study presents a rapid approach to evaluate and compare local sustainability in mining and tourism areas in Yunnan, China. The analysis was designed to employ currently available data in economic, social and environmental dimensions and included indicators of GDP, poverty, water quality, forest coverage, comprehensive ecological index, gender ratio and accessibility to roads and pipe water. It required minimum resources while still providing important insights on local sustainability. Our results indicate that mining areas performed better than tourism areas in economic aspects, but fell behind in social development, especially regarding the issue of gender balance. However, results from the t test show that differences between mining and tourism areas (except for gender ratio) are not significantly different than variations within mining or tourism areas.

Our study demonstrates that rapid SD evaluation can be used to compare sectoral performance and highlight areas that need attention from policy makers, agencies and academia. Although our analysis was limited by data availability and did not provide a comprehensive assessment on the outcomes, process and participation of SD, its simplicity makes it a useful tool for local government and agencies that are eager to understand local sustainability but limited by resources.
